# Interpersonal violence moderates sustained-transient threat co-activation in the vmPFC and amygdala in a community sample of youth

**DOI:** 10.1017/S0954579424001743

**Published:** 2024-11-26

**Authors:** Nadia Bounoua, Jane E. Joseph, Zachary W. Adams, Kathleen I. Crum, Christopher T. Sege, Lisa M. McTeague, Greg Hajcak, Colleen A. Halliday, Carla Kmett Danielson

**Affiliations:** 1 Department of Psychiatry, Medical University of South Carolina, Charleston, SC, USA; 2 Department of Neuroscience, Medical University of South Carolina, Charleston, SC, USA; 3 Department of Psychiatry, Indiana University School of Medicine, Indianapolis, IN, USA; 4 Department of Psychology, Florida State University, Tallahassee, FL, USA

**Keywords:** Childhood adversity, RdoC, threat processing

## Abstract

The increased risk for psychopathology associated with interpersonal violence exposure (IPV, e.g., physical abuse, sexual assault) is partially mediated by neurobiological alterations in threat-related processes. Evidence supports parsing neural circuitry related to transient and sustained threat, as they appear to be separable processes with distinct neurobiological underpinnings. Although childhood is a sensitive period for neurodevelopment, most prior work has been conducted in adult samples. Further, it is unknown how IPV exposure may impact transient-sustained threat neural interactions. The current study tested the moderating role of IPV exposure on sustained vmPFC-transient amygdala co-activation during an fMRI task during which threat and neutral cues were predictably or unpredictably presented. Analyses were conducted in a sample of 212 community-recruited youth (M/SD_age_= 11.77/2.44 years old; 51.9% male; 56.1% White/Caucasian). IPV-exposed youth evidenced a positive sustained vmPFC-transient amygdala co-activation, while youth with no IPV exposure did not show this association. Consistent with theoretical models, effects were specific to unpredictable, negative trials and to exposure to IPV (i.e., unrelated to non-IPV traumatic experiences). Although preliminary, these findings provide novel insight into how childhood IPV exposure may alter neural circuity involved in specific facets of threat processing.

## Introduction

Exposure to adverse childhood experiences is a potent predictor of a myriad of poor outcomes, including a range of psychopathologies and trauma-related problems (Adams et al., 2016; De Bellis, 2001; Green et al., 2010; Kessler et al., 2010; Molnar et al, 2001). Alterations to neural circuitry, particularly in neural circuits involved in negative emotional processes (e.g., threat processing), have been put forth as a key mechanism of psychopathology risk transmission among maltreated individuals (McEwen, 2007; McLaughlin & Lambert, 2017; Teicher et al., 2003). Specifically, accumulating evidence posits that links between the ventromedial prefrontal cortex (vmPFC) and amygdala, both of which are heavily involved in emotion regulation and threat detection processes, may be disturbed following childhood maltreatment (e.g., Dillon et al., 2014; Peverill et al., 2019). Further, it is posited that this neural circuit may be particularly sensitive to experiences characterized by a high degree of threat, such as interpersonal violence (IPV) (McLaughlin & Lambert, 2017). In the adult literature, recent work supports the need to disentangle the neural circuits involved in acute (e.g., fear) versus sustained (e.g., anxiety) threat processing to better elucidate how these systems interact to process threatening stimuli (Somerville et al., 2013). However, it remains unknown how threatening experiences in childhood influence vmPFC-amygdala activation patterns in relation to distinct facets of threat processing. The objective of this study is to address this gap in a large community sample of children and adolescents who completed a mixed-block design fMRI task that allowed for the simultaneous specification of both transient and sustained threat.

### Ventromedial prefrontal cortex & amygdala: links with threat processing

Based on extant meta-analytic work, the vmPFC and the amygdala have been consistently linked to threat processing (Colich et al., 2020; Webler et al., 2021). Contemporary research has shown that the amygdala is involved in the learning and detection of relevant and emotionally salient environmental stimuli to inform physiological and behavioral responses (Hariri, 2009; Ledoux, 2000; Lindquist et al., 2012). The vmPFC has been implicated in an array of emotional processing functions, including the regulation of emotional responses through projections to and from the amygdala (e.g., Dixon et al., 2017). As a circuit, it is posited that there are inhibitory bidirectional effects between vmPFC and amygdala activation, leading to an inverse relationship between activation levels in these regions during emotion regulation, broadly defined (Dixon et al., 2017; Ghashghaei et al., 2007; Ochsner et al., 2004; Urry et al., 2006). This inverse vmPFC-amygdala coupling is also present during threat detection, learned fear extinction, and fear generalization (Etkin et al., 2006; Greenberg et al., 2013a; Milad & Quirk, 2002; Morris & Dolan, 2004; Phelps et al., 2004). Further, research has shown this downregulation of amygdala by vmPFC has been linked to adaptive diurnal cortisol patterns, indicative of better general well-being (Urry et al., 2006). Along those same lines, altered amygdala-vmPFC connectivity is commonly linked to psychopathology across the life span, including anxiety disorders and posttraumatic stress disorder (Cisler et al., 2013; Etkin & Wager, 2007; Gold et al., 2016; Greenberg et al., 2013b; Koenigs & Grafman, 2009; Pagliaccio et al., 2015).

### Parsing acute and sustained threat processing

To date, the majority of literature has applied a broad conceptualization of “threat processing,” potentially obfuscating neural circuits involved in distinct threat-related processes. Research is needed that disentangles acute and sustained threat. Such an approach is consistent with the National Institute of Mental Health Research (NIMH) Domain Criteria (RDoC; Insel et al., 2010). Research shows that acute threat processing (e.g., fear) systems mediate momentary reactions to threat stimuli (or threat-predicting stimuli), whereas sustained threat systems (e.g., anxiety) mediate longer-lasting reactions when threat is more chronic (be it actually present or merely threatened) (Insel et al., 2010). Importantly, sustained threat activation can be differentiated from those changes evoked by transient threat. In a study of 55 psychologically healthy adults with the same task utilized here, Somerville and colleagues (2013) found support for dissociable neural circuits associated with these two dimensions of threat responding, such that amygdala showed transient response to negative stimuli (i.e., fear), while regions of the vmPFC were observed in relation to sustained threatening stimuli. Furthermore, authors found significant interactions between threat dimensions such that *sustained* vmPFC activation was negatively correlated with *transient* amygdala activation (Somerville et al., 2013). This inverse activation pattern is consistent with the hypothesized top-down regulatory control of the vmPFC on acute fear responding in the amygdala.

### Interpersonal violence as a moderating environmental factor

Previous work has shown that the vmPFC-amygdala circuit is highly sensitive to the effects of stress, particularly during the transition into adolescence (Pechtel and Pizzagalli, 2011). This neurodevelopmental period is marked by a high degree of neuroplasticity, especially in neural circuits involved in emotion-related processes (Tottenham & Galván, 2016). Emerging work highlights that different forms of childhood adversity exert distinct neurobiological disruptions, particularly in threat-related systems (McLaughlin & Lambert, 2017; McLaughlin & Sheridan, 2016). Interpersonal trauma exposure (i.e., violence, physical abuse, sexual assault, neglect), as opposed to other non-interpersonal forms of trauma exposure (e.g., natural disasters, car accidents, serious illness) has been uniquely linked to threat-related neural alterations, such as exaggerated neural responses to threatening stimuli (McCrory et al., 2013; McLaughlin et al., 2015). Importantly, these alterations reflect initially adaptive modifications in the context of threatening environments (McLaughlin & Lambert, 2017). More specifically, children exposed to repeated threatening events may develop enhanced threat detection processes that facilitate quick responses to danger and mobilize safety behaviors (McEwen & Gianaros, 2011). Over time, however, IPV-related experiences have been linked to chronic activation of stress response systems (Doom & Gunnar, 2013; Heim et al., 2008), which may lead to greater amygdala hyperactivity to threat (McCrory et al., 2013; McLaughlin et al., 2015). Therefore, IPV may be a form of child adversity that has particular influence on the vmPFC-amygdala circuit.

Neuroimaging studies have sought to clarify how exposure to childhood adversity impacts the vmPFC-amygdala circuit. At a neuroanatomical level, research has shown that the structural size (i.e. cortical thickness and/or volume) of amygdala and regions of the vmPFC are inversely correlated in healthy adults, thought to reflect the inverse functional relation described above (Albaugh et al., 2013; Blackmon et al., 2011). Childhood trauma moderated bilateral OFC–amygdala volumetric associations in a sample of adults (Bounoua et al., 2022). Specifically, adults with childhood trauma exposure showed a positive association between medial OFC and amygdala volume, whereas adults with no childhood exposure showed the negative OFC–amygdala structural association observed in prior research with healthy samples. Studies using resting-state fMRI methods have also found that childhood maltreatment was associated with lower amygdala-vmPFC connectivity, indicative of less of the expected top-down control (Hanson et al., 2019; Herringa et al., 2013; Nooner et al., 2013; Thomason et al., 2015). Similar alterations in vmPFC-amygdala circuitry have been observed in studies using task-based, emotion-related fMRI paradigms. For example, Peverill et al. (2019) found that, in a sample of 57 adolescents, vmPFC-amygdala connectivity when viewing negative images (compared to neutral images) was weaker among adolescents exposed to abuse than those without a history of maltreatment. During an emotional conflict task, Marusak et al. (2015) found that trauma-exposed youths (n = 14) exhibited reduced amygdala-PFC coupling, suggesting a failure to dampen amygdala reactivity when compared to matched comparison youth (n = 16). One potential explanation is that stress-exposed youth require a higher degree of prefrontal activation to successfully regulate threat-induced amygdala reactivity, consistent with findings from McLaughlin et al. (2015).

### Current study

Accumulating evidence implicates the vmPFC-amygdala circuit in threat detection processes, a circuit that appears to be particularly impacted by childhood adversity marked by high degrees of threat. Emerging research with adults has found that vmPFC-amygdala coupling can also be observed when parsing transient and sustained threat, potentially indicative of in-the-moment downregulation of amygdala reactivity to negative stimuli by the vmPFC. However, the extent to which i) this association is present in younger samples and ii) these associations may be altered among IPV-exposed youth remains relatively understudied. To address these gaps in the literature, the goal of the current study is to test whether the association between sustained vmPFC and transient amygdala activation to negative, unpredictable stimuli varies as a function of IPV. Based on previous literature examining alterations to vmPFC-amygdala coupling reviewed above, we expected that non-IPV-exposed youth would demonstrate the expected inverse association between vmPFC and amygdala (indicative of intact coupling), while IPV-exposed youth would demonstrate a positive vmPFC-amygdala association (indicative of disrupted coupling).

## Methods

### Participants

Participants were drawn from a larger Charleston Resiliency Monitoring Study (PI Danielson: R01MH112209), an accelerated, longitudinal cohort design study assessing threat processing across levels of analysis. Participants were recruited through advertisements in schools, pediatric clinics, and the general community. Participants were eligible if they were enrolled in third, sixth, or ninth grade, were between the ages of 7 and 16, and had an adult caregiver willing to participate. Exclusion criteria included monolingual non-English-speaking status, history of psychosis, or evidence of developmental delay or functional impairment that would interfere with completing study procedures. For the present study, youth with MRI contraindications (e.g., irremovable metal in the body) were also excluded. Of the 258 participants with usable neuroimaging data^1^, 46 participants were missing data on key study variables.

Thus, for the present study, the final sample of the current study consisted of 212 youth and their caregivers. Sample characteristics can be found in Table 1. Participants were M/SD_age_= 11.77/2.44 years old (age range: 8–16 years old; 51.9% male). Approximately half (56.1%) of the sample self-identified as White, with 33.2% of youth identifying as Black or African-American, 7.3% identifying as multiracial, and 3.4% endorsing another race. 10.2% of the youth identified as Hispanic/Latinx.

## Measures

### Exposure to interpersonal violence (IPV)

The UCLA Posttraumatic Stress Disorder Reactivity Index (UCLA-PTSD-RI for DSM 5; Steinberg et al., 2013) was administered as an interview to both children and their caregiver to assess for a variety of traumatic events. IPV was defined as exposure to any of the following 12 events: community violence, domestic violence, school violence, physical assault, sexual abuse, physical abuse, sexual assault, kidnapping/abduction, terrorism, war/political violence, forced displacement, or trafficking/sexual exploitation. Youth were categorized (Yes/No) as having experienced IPV if they and/or their caregiver reported that the child was directly victimized by or witnessed one or more of these events. We also assessed for exposure to non-violent forms of trauma, which included natural/manmade disasters, serious accidental injury, serious illness/painful and/or frightening medical procedures, neglect, the presence of impaired caregiver, bereavement, and caregiver separation.

### Unpredictable threat functional MRI (fMRI) task

The fMRI task was an adaptation of an experimental paradigm by Somerville and colleagues (2013). The task used a mixed-block-event-related design, and modeled brain response to pictures of valence (negative vs. neutral) and temporal predictability (predictable vs. unpredictable onset). At the beginning of each block, a written cue describing trial type is presented for 3000 ms: Predictable Negative, Predictable Neutral, Unpredictable Negative, and Unpredictable Neutral. In predictable blocks, a ticking clock appeared on the screen until the clock hand reached the 12 o’clock position. A picture was then presented on screen for 3000 ms. During unpredictable blocks, the clock did not advance in a meaningful way, and thus, the picture presentation randomly occurred. Between blocks, a crosshair was presented on screen for 1500ms. Pictures were selected from the International Affective Picture System (IAPS; Lang et al., 2008) that were threatening or neutral in nature (for details of the IAPS stimuli used in this study, please see Supplementary Table S2 in Huggins et al., 2022). Participants completed three runs of the task, each run consisting of one block of ten trials for each of the four conditions. Block order varied across task runs, and runs were counterbalanced across participants. Participants were instructed to press a button whenever they saw a picture appeared to encourage engagement. EPrime 3.0 software (Psychology Software Tools, 2012) was used to present task stimuli and record behavioral responses.

## Procedure

All study procedures were approved by the Institutional Review Board. Participants and their caregivers provided written informed consent/assent prior to data collection. Caregivers and youth participants provided demographic information and completed a battery of self-report questionnaires and behavioral tasks. Eligible youth also completed the experimental paradigm of predictable and unpredictable threat during an fMRI scan (see task description above). A metal screening questionnaire was completed by each participant and reviewed by the MRI technician prior to entering the MRI scanner. Participants were placed on the scanner bed and given ear protection, headphones, and head cushioning.

### MRI data acquisition

Images were acquired on a Siemens MAGNETOM PRISMA 3T MRI scanner (Erlangen, Germany) with a 32-channel head coil. Functional T2*-weighted images were acquired in an axial orientation and simultaneous multislice pulse sequence (acceleration factor of 3) with the following parameters: repetition time (TR)/echo time (TE) = 1100ms/30 ms; flip angle = 65 degrees; field-of-view (FOV) = 192 mm; voxel size = 3 mm isometric. A high-resolution T1-weighted magnetization-prepared rapid acquisition with gradient echo (MPRAGE) structural scan was acquired for co-registration with the functional data using the following parameters: TR/TE = 2300ms/2.26 ms; flip angle = 8 degrees; FOV = 256 mm; voxel size = 1 mm isometric. To correct for geometric distortions caused by static-field inhomogeneity, field maps were also collected.

## Data analysis

### fMRI preprocessing and individual-level statistics

fMRI data processing was carried out using FEAT (FMRI Expert Analysis Tool) Version 6.00, part of FSL (FMRIB’s Software Library) and included motion correction using MCFLIRT (Jenkinson 2002); non-brain removal using BET (Smith 2002); B0 field unwarping using FUGUE, spatial smoothing using a Gaussian kernel of FWHM 6 mm; grand-mean intensity normalization of the entire 4D data set by a single multiplicative factor; high-pass temporal filtering (Gaussian-weighted least-squares straight line fitting, with sigma = 115.0s). Registration to high-resolution structural and standard space images was carried out using FLIRT (Jenkinson 2001, 2002). Each usable functional run time-series statistical analysis was carried out using FILM with local autocorrelation correction (Woolrich 2001). One GLM was used which included eight explanatory variables for each of the four experimental conditions (PNEU, PNEG, UNEU, UNEG) modeled as single trials lasting 3 s each (transient response) and an entire block lasting 90 s each (sustained response), convolved with a double-gamma hemodynamic response function. The cue that preceded each block was also modeled as an EV lasting 3 s. Head motion outliers and six parameters of head motion were added as covariates. Temporal derivatives and temporal smoothing were applied to each of the task condition and the cue EVs. Contrasts of parameter estimates (copes) were generated for each run for each participant. Each of the usable runs for a participant were averaged using fixed effects analysis in FEAT and the copes from the averaged runs were submitted to voxel-wise group statistical analysis. For participants with only one usable run, the copes from the single run were used in the group analysis. The majority of participants had complete usable runs (74.1%), followed by two usable runs (18.4%), and one useable run (7.5%). Preliminary analyses revealed no group differences in key study variables (i.e., IPV exposure, amygdala and vmPFC activation) between youth with one, two, or three usable runs (all *p*’s > .05). Thus, all participants were retained in the data analysis.

Given our a priori regions of interest (ROI), we extracted average BOLD activation levels in the amygdala and vmPFC across these conditions. The vmPFC and amygdala ROIs were created as 5 mm-radius spheres that were centered on regions reported by Somerville et al. (2013) (peak coordinates left for vmPFC: −6, 37, −17; right vmPFC: 3, 29, −12; left amygdala: −21, −7, −17; right amygdala: 24, −1, −20) (see Figure S1 in Appendix). Given we did not have lateralization hypotheses, we opted to average activation across hemispheres for each ROI prior to analysis for a more parsimonious data analytic plan^2^.

Pearson correlations were used to assess bivariate relations among key study variables. Primary analyses were conducted using linear regression analyses, with standardized coefficients and confidence intervals provided as measures of effect size. First, a linear regression was conducted with sustained vmPFC activation predicting transient amygdala activation during unpredictable negative trials. Next, an interaction term between vmPFC activation and IPV exposure (Yes/No) was introduced to the regression to test for IPV moderation. Youth sex assigned at birth (male = 1; female = 2) and age were entered as covariates of no interest. Youth anxiety symptoms (measured by the Multidimensional Anxiety Scale for Children-2nd edition (MASC-2; March, 2013) were included as a covariate to ensure observed effects were not confounded by concurrent threat-related psychopathology (Hart & Rubia, 2012), consistent with similar neuroimaging work with maltreated youth (e.g., Lambert et al., 2017). All analyses were conducted using SPSS Version 28.

## Results

### Prevalence of trauma exposure

39% of youth reported experiencing at least one type of IPV in their lifetime. The most common form of violence exposure was school-related violence (39.5%), followed by exposure to domestic (32.1%) and community violence (21.3%), sexual abuse (15.0%), physical assault (14.8%), physical abuse (11.3%), and sexual assault (6.3%). No youth in this sample endorsed experiences of kidnapping, terrorism, war violence, or sexual exploitation/trafficking. There was a high degree of non-IPV forms of trauma present in our sample, including bereavement (66.3%), serious illness/medical trauma (46.2%), serious accidental injuries (39.5%), caregiver separation (35.0%), natural disasters (5.7%), neglect (10.0%) and the presence of an impaired caregiver (12.5%).

There was a high degree of overlap between IPV and non-IPV exposure, with approximately 35.1% of the sample reporting experiencing at least one form of IPV and non-IPV traumatic events. Further, among youth who denied IPV exposure, approximately 77.7% of these youth did report exposure to non-IPV trauma.

### Preliminary analyses

Bivariate associations among key study variables are presented in Table 2. Youth age and biological sex were weakly correlated, such that female youth tended to be older than male youth. Female sex was also weakly associated with greater youth-reported anxiety symptoms (*MASC-2*).

### Sustained-transient interactions in vmPFC – amygdala co-activation

First, we tested for the association between sustained vmPFC activation and transient amygdala activation during unpredictable, negative trials, after controlling for covariates. Results of the linear regression model can be found in Table 3. Age was significantly related to transient amygdala activation in the negative, unpredictable condition, such that older youth evidenced less amygdala activation. However, there was no significant association between sustained vmPFC and transient amygdala activation.

### Impact of IPV on sustained vmPFC – transient amygdala co-activation

**Figure 1.**
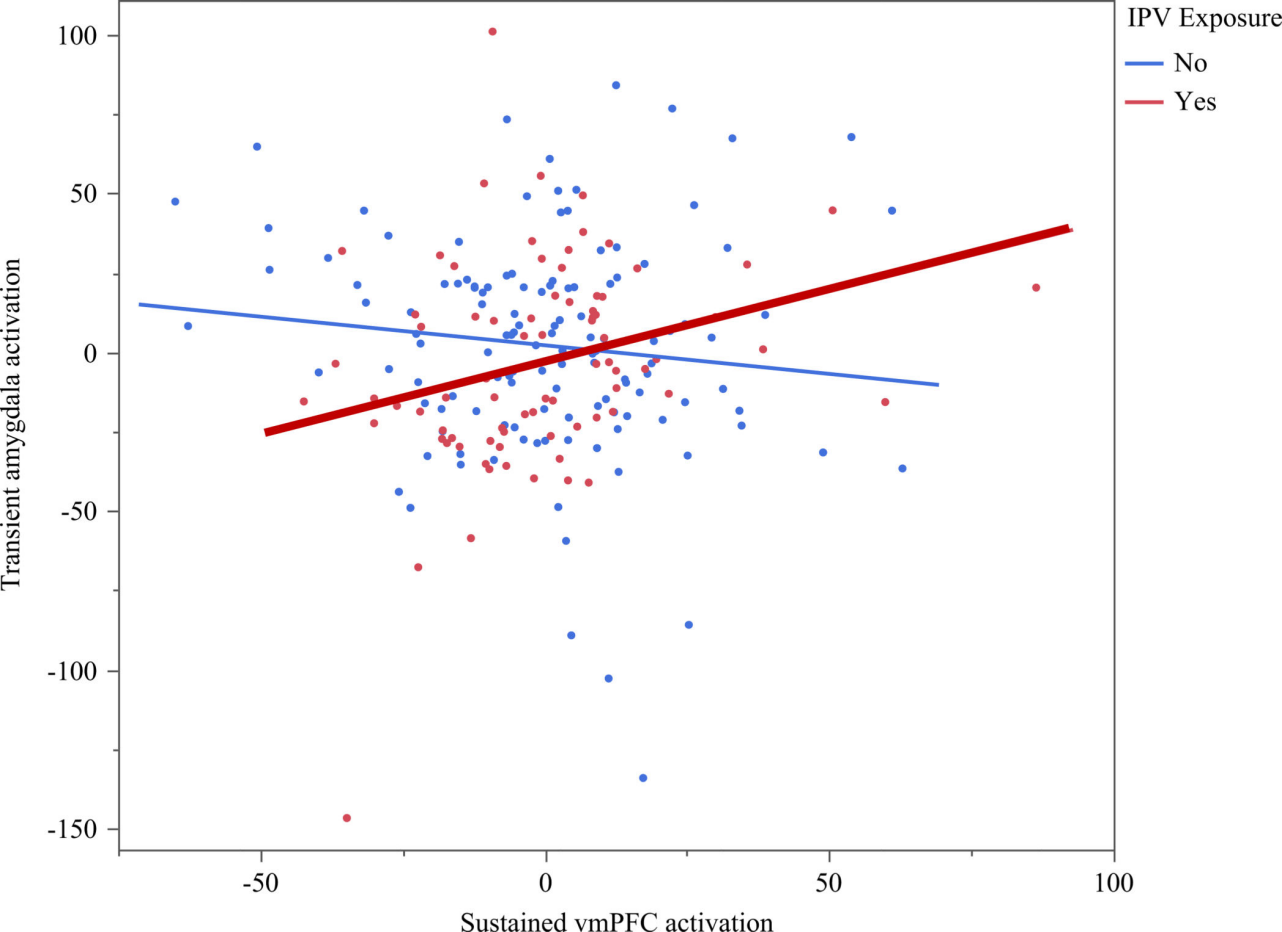
Exposure to IPV moderates vmPFC-amygdala associations during sustained vmPFC and amygdala transient threat responding to unpredictable negative stimuli. vmPFC = ventromedial prefrontal cortex; IPV = interpersonal violence. Bolded line indicated significant simple slope. 95% confidence intervals bands depicted.

Next, we tested whether the association between sustained vmPFC and transient amygdala activation during unpredictable, negative trials varied as a function of exposure to IPV. Results of the moderation analyses can be found in Table 4. There was a significant interaction effect (*p* = 0.012). Post hoc probing of the interaction revealed a significant, *positive* association between sustained vmPFC and transient amygdala activation among IPV-exposed youth (*t* = 2.24, *p* = 0.026, *95%CI* = 0.05/0.77), while no association was observed for youth with no IPV exposure (*t* =−1.20, *p* = 0.230, *95%CI* = –0.43/0.10). The significant interaction is depicted in Figure 1.

### Additional analyses

#### Specificity of negative, unpredictable condition

Our a priori hypotheses pertained to vmPFC-amygdala relations during negative and unpredictable trials, based on previous work (e.g., Somerville et al., 2013). To test whether the observed effects were specific to these conditions, we re-ran the above analyses using vmPFC-amygdala activation during other task conditions (e.g., neutral stimuli, predictable threat). We found no significant effects when examining sustained vmPFC – transient amygdala in unpredictable neutral condition or predictable negative condition (*p*’s>0.05), suggesting that the effect of IPV exposure on sustained vmPFC – transient amygdala co-activation was specific to negative and unpredictable trials.

#### Specificity of IPV vs. non-violent forms of trauma exposure

Our a priori hypotheses pertained to IPV forms of trauma given research suggesting that exposure to violent or threatening stressors in particular are associated with threat detection alterations (e.g., McLaughlin & Sheridan, 2016). We ran a supplementary analysis to test whether exposure to only non-violent forms of trauma (e.g., bereavement, illness) revealed similar moderating effects. Approximately half (n = 101; 47.6%) of the sample endorsed experiencing non-violent forms of trauma (and no IPV). The analysis revealed that exposure to non-IPV trauma did not moderate vmPFC-amygdala relations in the unpredictable, negative condition (*p* = 0.554), suggesting the observed effects may be particularly driven by violent forms of trauma exposure.

#### Developmentally relevant individual differences

Given research citing developmental differences (e.g., Gee et al., 2022; Tottenham & Galván, 2016) and sex differences (e.g., Bale & Epperson, 2015) in the development of neural circuits related to threat processing, we conducted supplementary analyses to test whether age or sex differences emerged in the role of IPV on vmPFC-amygdala activation patterns. First, we tested a 3-way interaction to determine whether there were age differences in the observed IPV moderation effect and found no significant age moderation (*t* = –0.99, *p* = 0.322, *95%CI* = –0.28, 0.09). Similarly, when testing for potential sex differences in the IPV moderation effects, we did not find a significant interaction (*t* = –.02, *p* = 0.987, *95%CI* = –0.45, 0.45).

## Discussion

Extant research has underscored the deleterious impact of IPV exposure on an array of physical, social, and mental health outcomes. These effects are particularly problematic early in life, as neurobiological systems are undergoing vast developmental changes that create heightened sensitivity to environmental stressors, including childhood trauma exposure. The vmPFC-amygdala circuit has been a well-studied neural correlate of threat processing across samples, with several existing studies documenting alterations to this circuit as a function of childhood adversity. However, threat processing represents a broad construct that can be parsed into meaningful subcomponents at the neurobiological level. The current study sought to extend on previous studies by examining whether childhood IPV exposure moderated vmPFC-amygdala associations during a novel adaptation of an fMRI task that parsed transient and sustained threat processing in youth. Consistent with previous research, we found that sustained vmPFC-transient amygdala activation to negative (but not neutral) stimuli differed as a function of IPV exposure, such that IPV-exposed youth displayed a positive association between sustained vmPFC and transient amygdala activation, potentially indicative of reduced inhibitory control of the vmPFC to amygdala activation. Although preliminary, these findings provide compelling evidence that IPV exposure may impact neural systems involved in distinct threat-related processes in youth.

Given work implicating neurobiological alterations as mechanisms of risk transmission between IPV and later psychosocial problems, the transition from childhood to adolescence represents a critical developmental period for studying neural correlates of threat processing before problematic trajectories have crystallized (Gee et al., 2022; Sisk & Gee, 2022). The vmPFC-amygdala circuit has been widely studied in the context of stress exposure, with numerous studies revealing alterations to this circuit at a neuroanatomical and functional level following trauma exposure. There is ample evidence of the bidirectional and inhibitory nature of vmPFC and amygdala activation, reflecting greater vmPFC activation in order to successfully down-regulate amygdala activation and promote well-being (Dixon et al., 2017; Ghashghaei et al., 2007; Ochsner et al., 2004; Urry et al., 2006). While the vmPFC-amygdala circuit has been consistently linked to various aspects of fear learning/processing, more work is needed to understand how stress exposure may impact this circuit during specific facets of fear processing. Specifically, there is evidence that among healthy adults, transient and sustained threat-related processing show different neural correlates in fMRI tasks (e.g., Somerville et al., 2013). However, the extent to which sustained-transient interactions in the vmPFC-amygdala circuit occur in youth, or the impact of IPV exposure on these associations, has not yet been investigated.

The first aim of this study was to examine the association between vmPFC activation to sustained threat and amygdala activation to transient responses in a sample of stress-exposed youth. Based on the existing neuroimaging work, we expected to find an inverse association between transient amygdala and sustained vmPFC activation to negative stimuli. Interestingly, we did not find a significant association between sustained vmPFC and transient amygdala activation in our sample of children and adolescents. This was somewhat surprising, considering Somerville and colleagues (2013) observed an inverse vmPFC-amygdala sustained-transient interaction using a similar task. One important difference that may explain these disparate findings is the difference in developmental stages between the samples of the two studies, as the Somerville et al. (2013) study included a sample of healthy *adults*. As noted above, the vmPFC-amygdala circuit undergoes tremendous neurodevelopment across the life span, with rapid changes occurring during early adolescence. Thus, we may expect to find different patterns of vmPFC-amygdala activation related to transient and sustained threat across developmental periods, particularly if youth in the sample are still undergoing cortical development of inhibitory circuits.

At the same time, numerous studies to date have shown that stress exposure plays an impactful role on the vmPFC-amygdala circuit, particularly when it occurs early in development (Tottenham & Galván, 2016). Thus, the second aim of the study was to test whether IPV exposure altered the association between sustained and transient threat processing in the vmPFC-amygdala circuit. Consistent with existing literature, we would expect that non-IPV-exposed youth would evidence more normative inverse vmPFC-amygdala activation associations, while IPV-exposed youth would evidence a positive vmPFC-amygdala association (indicative of less top-down control). We found partial support of our hypothesis. Specifically, we found that IPV exposure moderated the association between sustained vmPFC and transient amygdala activation, such that IPV-exposed youth evidenced a positive association between these regions, while non-IPV-exposed youth did not (see Figure 1). Interestingly, the non-IPV-exposed group did not show a significant vmPFC-amygdala association, although there was a trend in the expected negative direction. Based on the interpretations provided by Somerville et al. (2013), the design of the fMRI task allowed for the parsing of sustained and transient neural activation, which can be used to provide an index of how sustained vmPFC activation may modulate in-the-moment transient amygdala reactions to negative stimuli. They found that greater sustained vmPFC activation negatively predicting transient amygdala activation among healthy adults. Extending from this work, our results suggest that, at least for youth, IPV exposure may disrupt the communication between these two regions during transient and sustained threat processing. This would be in line with existing neuroimaging work demonstrating weakened resting and task-based fMRI vmPFC-amygdala coupling among maltreated or stress-exposed youth (e.g., Herringa et al., 2016; Marusak et al., 2015; Peverill et al., 2019). Given research showing that the development of amygdala precedes the development of prefrontal regions and, subsequently prefrontal–limbic connectivity (Gee, Gabard-Durnam, et al., 2013; Gee, Humphreys, et al., 2013), one potential interpretation of these findings is that IPV-exposed youth may have experienced early stress-induced amygdala hyperactivity that leads to alterations in the development of amygdala-PFC connectivity as development continued (Tottenham & Galván, 2016). More research will be needed to further explore how and when IPV exposure impacts these circuits to further our understanding of the impact of stress exposure on neural circuitry supporting threat processes.

Although preliminary, the current findings reveal potentially useful insights into how IPV exposure may disrupt neural systems involved in threat processing during adolescence. For example, bivariate correlations revealed that IPV was not directly associated with transient amygdala nor sustained vmPFC activation, but rather modulated the co-activation between these regions as a function of transient and sustained threat activation during the fMRI task. Additionally, follow-up analyses revealed that the moderating effect of IPV on vmPFC-amygdala activation was specific to negative and unpredictable trials (but not neutral or predictable trials, regardless of valence), which suggests that IPV exposure may specifically impact co-activation during certain types of threat processing. One speculative interpretation of our findings is that IPV-exposed youth fail to sufficiently engage vmPFC during sustained unpredictable threatening contexts, which then prevents the dampening of transient amygdala activation to acute threatening presentations, thus leading to the observed positive association between these regions. Alternatively, it is possible that, among IPV-exposed youth, greater transient amygdala activation results in greater vmPFC activation that is sustained during threatening contexts. Thus, the positive vmPFC-amygdala association observed only among IPV-exposed youth may reflect an attempt at regulating emotional responses to the unpredictable and negative stimuli, but if the inhibitory circuit is compromised, more effort is required leading to greater vmPFC activation. Indeed, McLaughlin et al. (2015) found that maltreated adolescents were more likely to recruit prefrontal regulatory regions than controls to reach the same level of emotion regulation. This interpretation is consistent with recent conceptualizations of childhood adversity that posit neurobiological adaptations observed among stress-exposed youth may actually reflect short-term adaptations to threatening and/or unpredictable environments (e.g., Callaghan and Tottenham, 2016; McLaughlin & Lambert, 2017). More research, particularly with longitudinal data, will be needed to examine how sustained-transient interactions in threat processing in the vmPFC-amygdala circuit relate to concurrent and future functioning.

### Clinical implications

These findings may translate to further etiological models of psychopathology as they occur among trauma-exposed children and adolescents. Indeed, previous work has linked aberrant vmPFC-amygdala connectivity to an array of mental health problems, including anxiety and posttraumatic stress disorder (e.g., Cisler et al., 2013; Kim et al., 2011; Pagliaccio et al., 2015). In one study, Johnstone et al. (2007) found that, while nondepressed individuals showed an inverse vmPFC-amygdala relationship activation during an effortful affective reappraisal task, depressed individuals evidenced a positive vmPFC-amygdala association. In the current study, we found a similar positive vmPFC-amygdala relationship but only among IPV-exposed youth, even after accounting for anxiety symptoms, which may provide some insight into how trauma exposure may confer risk for concurrent and future psychopathology via threat-related neural circuitry. Ultimately, this line of research has the potential to inform interventions targeting stress-exposed youth, particularly by targeting threat-related neural circuitry (Gee et al., 2022; Lee et al., 2014).

### Strengths, limitations, & future directions

The current study had several strengths that advance the current literature. To our knowledge, this is the first study to investigate the impact of IPV exposure on sustained-transient vmPFC-amygdala co-activation in a youth sample. Additionally, we tested hypotheses in a relatively large diverse community sample of children and adolescents relative to existing studies, resulting in greater statistical power and generalizability of our findings. However, findings should be interpreted in light of some study limitations. First, our study used a cross-sectional design, which limited our ability to infer casualty of the effect of IPV, as well as delineate how timing of IPV exposure may impact vmPFC-amygdala circuitry across development (e.g., Cowell et al., 2015). Second, the UCLA-PTSD index used to assess for traumatic events is not comprehensive, and thus, other forms of IPV may not have been fully captured in this assessment. Further, this measure only provides a dichotomous indicator of exposure to each IPV type, and thus, we did not have the ability to examine how the severity (e.g., number of exposures) and chronicity of IPV may impact these results. Future studies replicating these findings with additional measures of IPV would address these gaps. Similarly, we did not examine how the severity and chronicity of IPV may impact these results. Studies using a longitudinal design would be useful in extending on these findings to better understand how and when IPV exposure impacts brain development. Third, we focused on the vmPFC and amygdala as ROI based on previous studies; however, it is likely that other brain regions relevant to threat processing may be impacted by IPV exposure. Thus, future research should extend on these findings by testing for effects in other brain regions. Further, while there are advantages to assuming an HRF shape, it is important to note that one limitation of this approach is that deviations from the double-gamma in the transient responses has the potential to mismodeled as a sustained response. Another approach would be to analyze these data without assuming a shape (e.g., FIR model), which may be less biased and reduce the likelihood of mismodeling of transient and sustained responses, but has trade-offs related to noise and power (e.g., Lindquist et al., 2009). Moreover, the present co-activation approach does not capture temporal synchrony between brain regions assessed within individuals, and the co-activation method used in this study does not enable investigation of the directionality of the influence of one region on another region. Advanced methods like dynamic causal modeling or Granger causality modeling could be used in future studies to address this. Finally, although we focused on IPV in this study, it is important to acknowledge the impact of other forms of stressors and social determinants of health (e.g., discrimination and race-related trauma, cumulative effects of poverty) on mental health and brain development (e.g., Bernard et al., 2021; Huggins et al, 2022). Thus, future work should aim to extend on these findings to further elucidate the impact of other forms of environmental stressors on neural correlates of threat processing.

## Conclusions

While a growing literature demonstrates the impact of childhood trauma exposure on neurodevelopment, more research is needed to better understand the mechanisms through which this risk is conferred. In the present study, we found that IPV moderated the co-activation between sustained and transient activation in the vmPFC and amygdala, respectively. To our knowledge, this is the first study investigating the role of IPV on neural circuits on related, yet separable, threat processes among youth. Findings have the potential to inform etiological models of psychopathology among youth with IPV, which is particularly important given that adolescence is a sensitive period for neurodevelopment and the emergence of psychopathology. Future work will be needed to replicate and extend on these findings to further our understanding of how childhood IPV exposure may alter neural circuity involved in specific facets of threat processing.

## Supplementary Material

1

## Figures and Tables

**Table 1.** Sample characteristics (*N* = 212)

Demographics	% of sample
Biological Sex	
Male	51.9
Female	48.1
Current Grade Level	
**3**^**rd**^	25.9
**6**^**th**^	34.9
**9**^**th**^	39.2
Youth Reported Racial Identity	
White	56.1
Black/African-American	33.2
Asian	1.0
Multiracial	7.3
Other	2.4
Youth Reported Ethnicity	
Hispanic	10.2
Non-Hispanic	89.8

**Table 2.** Bivariate correlations among study variables with 95% confidence intervals

	1	2	3	4	5	6
^1^ Youth age	––					
^2^ Sex Assigned at Birth	.20* **[.06, .33]**	––				
^3^ Youth Anxiety	−.11 [−.24, .03]	.17* **[.03, .29]**	––			
^4^ IPV Exposure	−.03 [−.17, .11]	.01 [−.12, .15]	.13 [−.01, .26]	––		
^5^ Transient Amygdala	−.18* **[−.31, −.05]**	−.03 [−.16, .11]	.08 [−.05, .21]	−.07 [−.20, .07]	––	
^6^ Sustained vmPFC	−.10 [−.24, .04]	−.07 [−.20, .07]	−.07 [−.21, .06]	−.02 [0.16, .11]	.04 [−.10, .17]	––

*Note. N* = 212. Significant correlations bolded. Sex assigned at birth: 1 = Male, 2 = Female. Youth anxiety was measured using the Multidimensional Anxiety Scale for Children – Second edition (MASC-2). IPV = interpersonal violence.

* *p*<0.05.

**Table 3.** Association between sustained vmPFC and transient amygdala reactivity across the entire sample (after controlling for covariates)

Model	B	SE	Standardized Beta	p-value	95% Confidence Interval for B	
					Lower bound	Upper bound
Age	−2.48	1.02	−.18	.016	−4.49	−.48
Biological Sex	.26	2.48	.01	.916	−4.63	5.16
Youth Anxiety	.09	.12	.06	.418	−.13	.32
Sustained vmPFC	.04	.11	.03	.696	−.18	.26

*Note. N* = 212. Sex assigned at birth: 1 = Male, 2 = Female. *R*^2^= .04.

**Table 4.** IPV exposure moderates the association between sustained vmPFC and transient amygdala reactivity (after controlling for covariates)

Model	b	SE	Standardized Beta	p-value	95% Confidence Interval for B	
					Lower bound	Upper bound
Age	−2.48	1.00	−.18	.014	−4.46	−0.51
Biological sex	.17	2.45	.01	.944	−4.65	5.00
Youth anxiety	.12	.11	.07	.304	−0.11	0.34
Sustained vmPFC	−.16	.14	−.10	.230	−0.43	0.11
IPV exposure	−8.47	4.98	−.12	.090	−18.28	1.34
**Sustained vmPFC × IPV exposure**	.57	.23	.22	.012	.13	1.02

*Note. N* = 212. Sex assigned at birth: 1 = Male, 2 = Female. IPV = interpersonal violence. *R*^2^= .07.

## Footnotes

1 Participants were excluded due to poor data quality (e.g., bad registrations, excessive movement, incomplete scanning data) or not having at least one usable run of data (out of three runs total).

2 Transient activation in right and left amygdala during unpredictable, negative trials were highly correlated (*r* = .78, *p* < .001 as was sustained activation in right and left vmPFC during unpredictable, negative trials (*r* = .62, *p* < .001).
