# Supplementary figures and images for: Interpersonal violence moderates sustained-transient threat co-activation in the vmPFC and amygdala in a community sample of youth
Source: Dev Psychopathol. Author manuscript; Available in PMC 2025 Sep 5. (PMC12104484; doi:10.1017/S0954579424001743)

**Appendix**

**Figure S1**. Location of ROIs


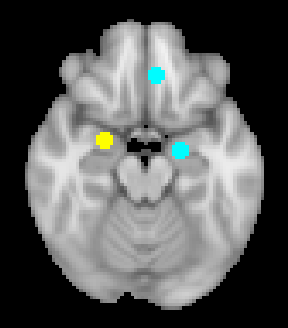

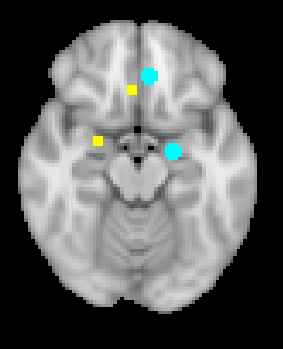

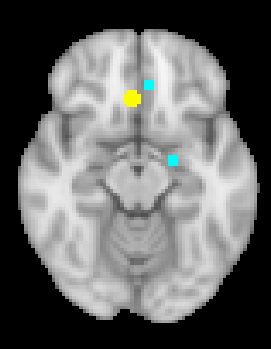


Z=-14

Z=-16

Z=-18

R

Supplement: 1 [file NIHMS2030682-supplement-1.docx]
